# Longitudinal Quantification of Eye-Movement Impairments after Pontine Hemorrhage

**DOI:** 10.3389/fneur.2017.00165

**Published:** 2017-05-02

**Authors:** Melis Suner, Glen T. Prusky, Jason B. Carmel, N. Jeremy Hill

**Affiliations:** ^1^Burke Medical Research Institute, White Plains, NY, USA; ^2^Blythedale Children’s Hospital, Valhalla, NY, USA; ^3^Weill Cornell Medicine, New York, NY, USA

**Keywords:** hemorrhage, pons, ophthalmoplegia, olivary nucleus, nystagmus, pathologic, eye tracking, computerized measurement

## Abstract

**Introduction:**

We report a case of hypertrophic olivary degeneration due to pontine hemorrhage. A 59-year-old male with untreated hypertension suffered a primary pontine hemorrhage, which caused horizontal eye-movement limitation. Progressive neurological deterioration with involuntary eye and palatal movements began months after hemorrhage. This was accompanied by magnetic resonance imaging evidence of hypertrophic olivary degeneration at 4.5 months.

**Background:**

Primary pontine hemorrhage often leads to impairment of eye movements and diplopia. Hypertrophic olivary degeneration can also emerge months after hemorrhage, producing involuntary pendular eye movements. Neither the natural history of voluntary eye movements nor the emergence of involuntary eye movements after pontine hemorrhage has been previously quantified.

**Methods:**

We used an optokinetic task that enabled measurement of eye movements. It provided real-time feedback on the ability to track continuously and saccade quickly in a pursuit task. The feedback motivated the patient to use the system repeatedly in his home. From 3 months after hemorrhage, the patient used the system for 9 months, allowing us to quantify changes in his eye movements.

**Results:**

Horizontal gaze impairments were manifest in our task as limitation in horizontal range of motion, as well as delay in initiation of the right eye’s movement during left-to-right pursuit. Improvement in these impairments was measured over the course of months 3–7 post hemorrhage. In addition, the emergence of vertical pendular nystagmus was identified in the subject at 4 months. Analysis of the eye-movement records revealed presymptomatic oscillatory eye movements whose amplitude had grown steadily over the course of 3 weeks, prior to a sharp increase in amplitude that coincided with the patient’s first report of oscillopsia. Horizontal pendular nystagmus emerged 7.4 months after the hemorrhage, primarily in the left eye.

**Conclusion:**

An eye-tracking system deployed in a patient’s home enabled prospective longitudinal quantification of the natural history and improvement in voluntary eye-movement impairments after pontine hemorrhage. It also characterized prospectively for the first time, the emergence of involuntary eye movements resulting from the rare complication of hypertrophic olivary degeneration. Results suggest that brief weekly measurements with an eye-tracker may allow early detection of this complication.

## Introduction

**Figure 1:**
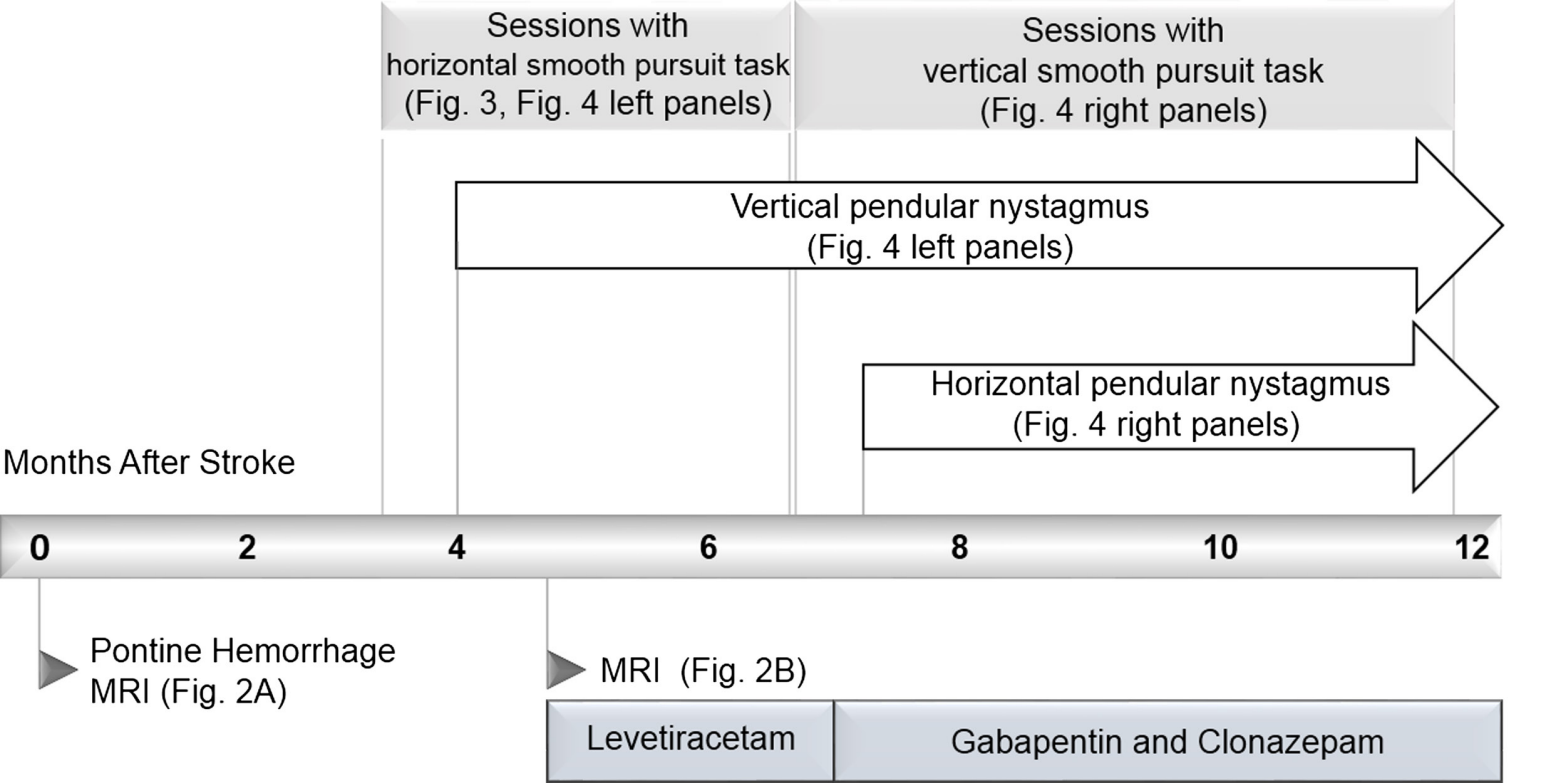
**Timeline of important events in the case with references to other Figures**.

We present a case of pontine hemorrhage resulting in bilateral ophthalmoplegia and the rare complication of hypertrophic olivary degeneration resulting in pendular nystagmus. The report is unique because we were able to make quantitative measurements of the patient’s eye movements regularly and repeatedly in his home. A novel eye-tracking system motivated the patient’s repeated participation by giving real-time feedback on an eye-movement task. This enabled us to record prospectively and in unprecedented detail, the recovery of gaze disorders and the emergence of secondary complications. The timeline of the case is summarized in Figure 1. The novel system and our quantitative analysis of its output are presented in Sections “Methods” and “Results,” respectively.

### Case History

#### Onset of Disease

**Figure 2:**
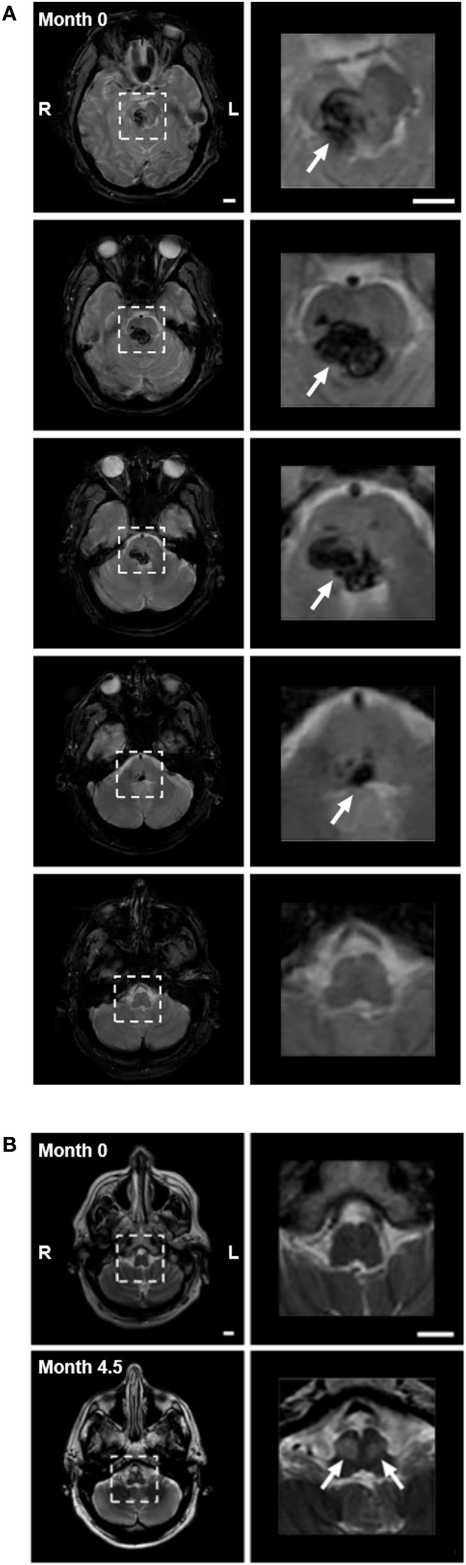
**(A)** Pontine hemorrhage imaged with gradient echo T2-weighted magnetic resonance imaging (MRI) within 24 h of hemorrhage. Left panels show consecutive 5-mm axial slices through the brainstem. Dashed squares highlight the region of hemorrhage. Right panels show 3.5× magnification of the highlighted region, with arrows indicating the hemorrhage (dark). The hemorrhage involved the pons and midbrain but not the medulla, primarily on the right side. **(B)** Axial images with turbo-spin echo T2-weighted MRI of medulla at time of hemorrhage (month 0—top panels), and after 4.5 months (month 4.5—bottom panels). Top panels: low (left) and higher (right) magnification images through the medulla (highlighted with dashed box) showed no evidence of hyperintensity at the time of hemorrhage. Bottom panels: bilateral focal hyperintensity of the anterior medulla at the level of the inferior olivary nucleus (arrows) was present at month 4.5, indicating the presence of hypertrophy. Scale bars = 1 cm.

A 59-year-old male Caucasian lawyer with untreated diabetes and hypertension suffered a sudden onset of vertigo, horizontal diplopia, and involuntary eye movements, without loss of consciousness. He presented to the ER with quadriplegia, impaired speech, ptosis of the right eyelid, and limited horizontal eye gaze. A magnetic resonance imaging (MRI) performed within 24-h revealed hemorrhage in the right pons and midbrain, with extension across the midline (Figure 2A). Involuntary eye movements subsided within 1 month of the hemorrhage.

#### Clinical Exam 3 Months after Hemorrhage

By 3 months after stroke, diplopia was the patient’s most impairing deficit and was not improving, despite his participation in inpatient rehabilitation. On primary gaze, the right eye was esotropic (this had resolved on reexamination at 7 months). The extent of movement with both eyes was limited when looking to the left or right. The right eye was more limited than the left in both adduction and abduction. Nystagmus was noted in the left eye during leftward gaze and in the right eye during rightward gaze (see Video S1 in Supplementary Material). Vertical and horizontal saccades were slowed. Within the angular limits imposed by his ophthalmoplegia, the patient was able to initiate and maintain smooth pursuit in both directions. Convergent eye movements were also preserved, and there was no evidence of facial palsy.

#### Development and Progression of Pendular Nystagmus

The patient reported a sudden onset of vertical oscillopsia and dizziness at 4 months. Clinical examination soon after revealed conjugate vertical pendular nystagmus (VPN) accompanied by palatal tremor. This prompted a second MRI at 4.5 months, which revealed no new hemorrhage or infarct, but instead showed hypertrophic degeneration of the inferior olivary nucleus (Figure 2B). In subsequent months, horizontal pendular nystagmus (HPN) also developed, and palatal tremor continued (see Video S1 in Supplementary Material). At 4.5 months, Keppra (levetiracetam) was prescribed at a dose of 1,000 mg per day. At 7 months, Keppra was replaced by Neurontin (gabapentin; 300 mg per day) and Klonopin (clonazepam; 0.5 mg per day)—see Figure 1. Neither medication abolished the nystagmus. The patient reported drowsiness while taking Neurontin and Klonopin.

## Background

Primary pontine hemorrhage accounts for 5–10% of intracranial hemorrhages in the US, with an incidence of 2–4 per 100,000 people each year (1). Affected patients present with diverse neurological deficits, including impaired eye movements, which are among the most severe and debilitating (1–3). Horizontal eye movements in particular are affected, since critical control circuitry is thought to be located in the pons (4). The limitation can vary from a complete loss of motion beyond the midline to a mild decrease in velocity or acceleration without any decrement in range of motion (5). If the oculomotor nucleus is also damaged (as with our subject—Figure 2A, top right), this may also contribute to the impairment of eye movements. Clinical assessments after damage to the pons rarely quantify the degree of such impairments and their recovery, and thus, we have limited information regarding the natural history of recovery and treatment efficacy.

Involuntary eye movements can develop months after pons damage. A rare example is the emergence of pathological nystagmus, with or without palatal tremor, long after injury (6). These impairments are associated with hypertrophic olivary degeneration, a secondary response to an interruption of afferent connections (7) in which inferior olive neurons and their nuclei become enlarged (5, 8, 9). Hypertrophic olivary degeneration can be the result of trauma, infection, demyelination (10), neoplasm, or vascular injury affecting the brain stem (11, 12). In addition to oculopalatal tremor, symptoms include gait ataxia, Holmes tremor (13), and change in the perception of gravity (7). The hypertrophy can be detected with imaging 4–6 months after the event and usually resolves within the first year, though pendular nystagmus and palatal tremor may persist for longer (9, 14, 15). The circuits underlying these involuntary movements are thought to be distinct from those subserving saccades, smooth pursuit, and the optokinetic and vestibulo–ocular reflexes (11). Therefore, saccades and smooth pursuit may remain intact in the presence of pendular nystagmus (16). Animal models and human pathology studies indicate that separate oscillators reside in the inferior olive for vertical, horizontal, and torsional movements (12, 17). However, animal models do not reproduce the characteristic human waveforms exactly (17), and the origins of the symptoms are not fully understood.

The natural history of eye-movement recovery from pontine hemorrhage and the acquisition of nystagmus with hypertrophic olivary degeneration have not been well characterized. This is due in part to the difficulty of repeatedly measuring ocular motor function in affected patients. In a clinical setting, examination is limited to qualitative observation and clinical judgment (18, 19); vertical and horizontal components of pendular nystagmus have previously been evaluated with electrooculography and video-based analysis (11, 20), but the condition has not previously been described prospectively. In a research setting, eye-tracking methods to quantify gaze and eye movements are difficult to calibrate accurately, expensive to implement, and require a technical team to administer. Thus, the lack of simple and objective measures of eye-movement function that can be administered in a home environment has made it difficult to follow the natural history of ocular motor disease, make predictions for recovery, and evaluate treatment efficacy.

## Methods

### Magnetic Resonance Imaging

At admission to ER, a Siemens 1.5 T Symphony MRI scanner was used to produce a gradient echo transverse-relaxation-time (T2)-weighted sequence, and a turbo-spin echo (TSE) T2-weighted sequence. TSE T2 imaging was repeated 4.5 months later with a GE 3 T MRI scanner. All sequences used 5-mm sections.

### Eye-Movement Testing System (“OptokineSys”)

A 27″ widescreen all-in-one computer was placed 62 cm from the patient’s eyes, with a viewable area subtending 51.3° horizontally and 30.3° vertically. A high-contrast 360° panoramic scene was scrolled horizontally across the screen at 12°/s or vertically at 6°/s. Eye movements were simultaneously recorded with a screen-mounted infra-red eye-tracker (Tobii Technology, Stockholm, Sweden) sampling at 60 Hz. The patient was instructed to follow the movement of the scene with his eyes from one edge of the screen to the other, with minimal head movement, and then perform a reverse saccade to reset and continue the process. The scene was composed such that there would be many salient details for the subject to fixate at any one time. Refractive correction was used during testing (the patient’s own contact lenses).

Software computed in real time whether the patient’s gaze was on the screen and quantified his ability to follow the stimulus continuously with his eyes. Whenever the patient’s gaze could not be measured adequately, the screen was tinted orange, which cued him to open his eyes wider, look at the screen, and/or correct his viewing distance. During task performance, an algorithm computed a score quantifying the concordance of his eye movements with stimulus motion—this was affected both by his limited range of motion and the slowing of his saccades. To encourage task participation, whenever the score surpassed a preset criterion, the system played music chosen by the patient; whenever the score fell below criterion, the music was paused. The score was smoothed in time such that it tolerated short interruptions from reverse saccades or blinks. The algorithm was based on real-time computation of a velocity vector: gaze position in one sample, minus its position in the most recent previous sample, divided by the time lag between the two samples. Since this computation relies only on relative *differences* between successive position estimates, it enables measurement without the need to determine absolute gaze position, which is particularly difficult to calibrate in a patient with eye-movement disorders. Thus, we did not spatially calibrate the eye-tracker to the subject but relied on a generic calibration of a male with a comparable interpupillary distance.

For the first 4 months, the patient used the system twice a week on average, for 25 min per session, with stimuli moving horizontally. From the onset of vertical nystagmus for about 5 months, he chose to use the system with vertically moving stimuli.

The patient gave written informed consent to participation in the study and, separately, to its publication as a case report.

### Analysis Methods

To maximize homogeneity of the data and allow for fair comparisons, most eye-movement metrics were computed only from sessions in which the patient tracked in a particular direction (for example, the patient performed left-to-right tracking sessions more regularly than right-to-left, so most of the horizontal analyses were restricted to left-to-right sessions). Since some sessions yielded only 2 min of tracking data of the desired type, analyses also were restricted to the first 2 min of the record.

#### Range of Eye Movements

We computed the average extent, in degrees, of the slow-phase sweeps from left to right during tracking. The statistic was computed for each eye and reflected horizontal gaze limitations.

#### Right-Eye Delay in Initiation of Tracking

This was the time difference, in milliseconds, between when the left eye began tracking smoothly and the right eye began tracking smoothly, averaged across all left-to-right tracking instances. This captured the patient’s pronounced right-eye movement limitation—whereas the left eye began its left-to-right slow tracking movements all the way to the left of the screen, the right eye could not reach this gaze position and only joined in the tracking part-way through each sweep.

#### Vertical Pendular Nystagmus

The VPN was computed using average peak-to-trough amplitude, in degrees, of involuntary oscillations in vertical gaze position. To ensure that vertical deviations did not arise from the patient scanning the scene looking for a visual feature to track, only the smooth phases of horizontal tracking were considered—periods in which saccades occurred were excluded. VPN was considered to be present if the amplitude of vertical oscillations in the 2–6 Hz range (20, 21) exceeded 0.5° in one eye (22, 23), or differed between eyes by more than 0.1°.

#### Horizontal Pendular Nystagmus

For HPN, the same calculation was used as for VPN, except that horizontal oscillations were assessed during vertical (downward) smooth tracking phases.

#### Consistency of Tracking

Consistency of tracking was computed as the total duration over which horizontal tracking score surpassed criterion (music played) as a percentage of the time that the patient’s eyes could be tracked (screen not orange). This is the output of the algorithm that compares patient’s eye-movement velocity with stimulus motion. For this analysis, all eye/direction conditions were considered, in the proportions they were performed according to the patient’s choice. The result reflected the degree of success the patient experienced with the system.

## Results

### Eye Movements Measured 3 Months after Hemorrhage

**Figure 3:**
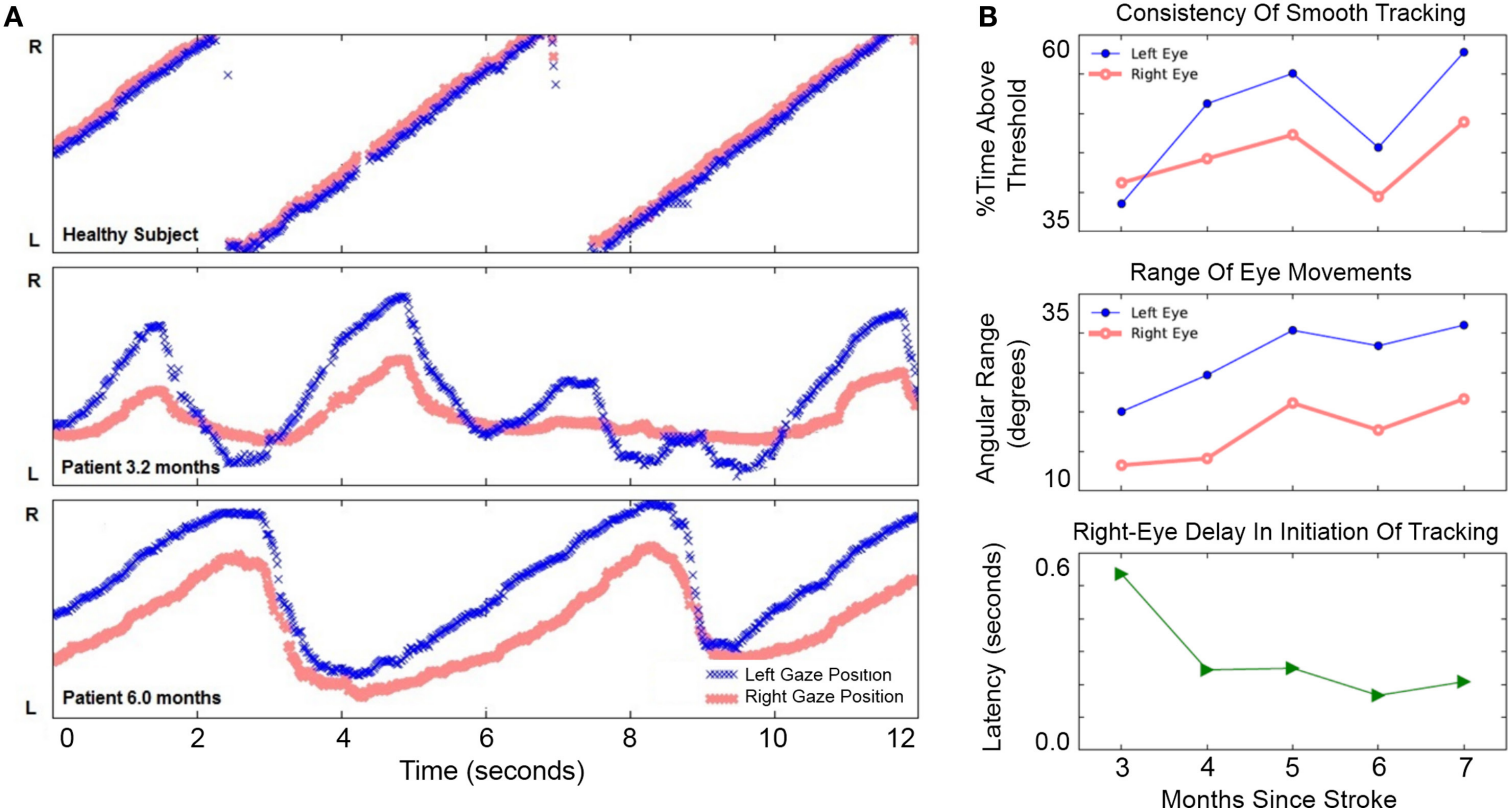
**Improvement of horizontal eye movements during left-to-right smooth pursuit**. **(A)** Horizontal gaze position during 12 s of left-to-right smooth pursuit interspersed with reverse saccades. The *Y*-axis in gaze position recordings spans 46° of the horizontal visual field. Top: representative horizontal gaze position in a healthy pilot subject. Middle: representative sample from session completed 3.2 months after hemorrhage when eye movements were not conjugate and saccades were slower than a healthy subject’s (compare with top panel). The right eye’s range of horizontal movement relative to the left eye was limited; the right eye initiated smooth rightward movement with a delay relative to the left eye. Bottom: representative sample from session completed 6 months after hemorrhage. Eye movements were more conjugate. Both eyes were able to perform longer sweeps of smooth pursuit and more-rapid reverse saccades. **(B)** Improvement of voluntary eye movements. Top: consistency of left eye movements (blue filled circles) may have improved more than right-eye movements (red open circles), although neither trend was significant (see text). Middle: range-of-movement was greater for the left eye than the right eye at 3 months. The range-of-movement of both eyes improved significantly from 3 to 7 months. Bottom: right-eye delay in initiation of rightward tracking was present at 3 months but improved significantly by 7 months.

The patient chose to work on left-to-right pursuit tasks the most, since these were most impaired—thus, most analyses are based on left-to-right tracking sessions. At 3.2 months after hemorrhage, the patient showed disconjugate gaze during fixation and pursuit. The right eye’s range of horizontal motion in both directions was more limited than the left (Figure 3A).

### Improvement in Horizontal Eye Movements

From 3 to 7 months after hemorrhage, the patient engaged in horizontal pursuit; he completed 192 sessions totaling 708 min during this period and reported improvement in diplopia. The lower panel in Figure 3A shows a measurement at 6 months: gaze was more concordant between left and right eyes than at 3.2 months, and both eyes performed pursuit over a greater range.

Improvement in horizontal eye movements from 3 to 7 months after hemorrhage is shown in Figure 3B. The consistency of tracking (Figure 3B, top) was 39% at 3 months and 58% at 7 months, but this apparent improvement was not statistically significant (Pearson’s *r* = 0.29, *p* = 0.158, *N* = 26). The smaller improvement in right-eye consistency, from 41 to 49%, was also not significant (*r* = 0.29, *p* = 0.219, *N* = 26). Range-of-movement during the horizontal task (Figure 3B, middle) was greater in the left eye than in the right throughout the period. From 3 to 7 months, the range-of-movement of both eyes improved significantly. The left eye improved from 20.1° to 31.0° (*r* = 0.58, *p* = 0.003, *N* = 24); the right eye improved from 13.3° to 21.7° (*r* = 0.69, *p* = 0.0002, *N* = 24). Initiation of smooth tracking motion in the right eye lagged behind the left eye by 536 ms at 3 months (Figure 3B, bottom). Over the first month of usage, the patient’s right-eye delay decreased sharply and significantly (*r* = −0.76, *p* = 0.007, *N* = 11). There was only slight improvement thereafter the delay was reduced to 208 ms by 7 months. Over the entire period, the correlation remained significant (*r* = −0.41, *p* = 0.048, *N* = 24). These results document improvement in horizontal gaze limitations.

### Pendular Nystagmus

**Figure 4:**
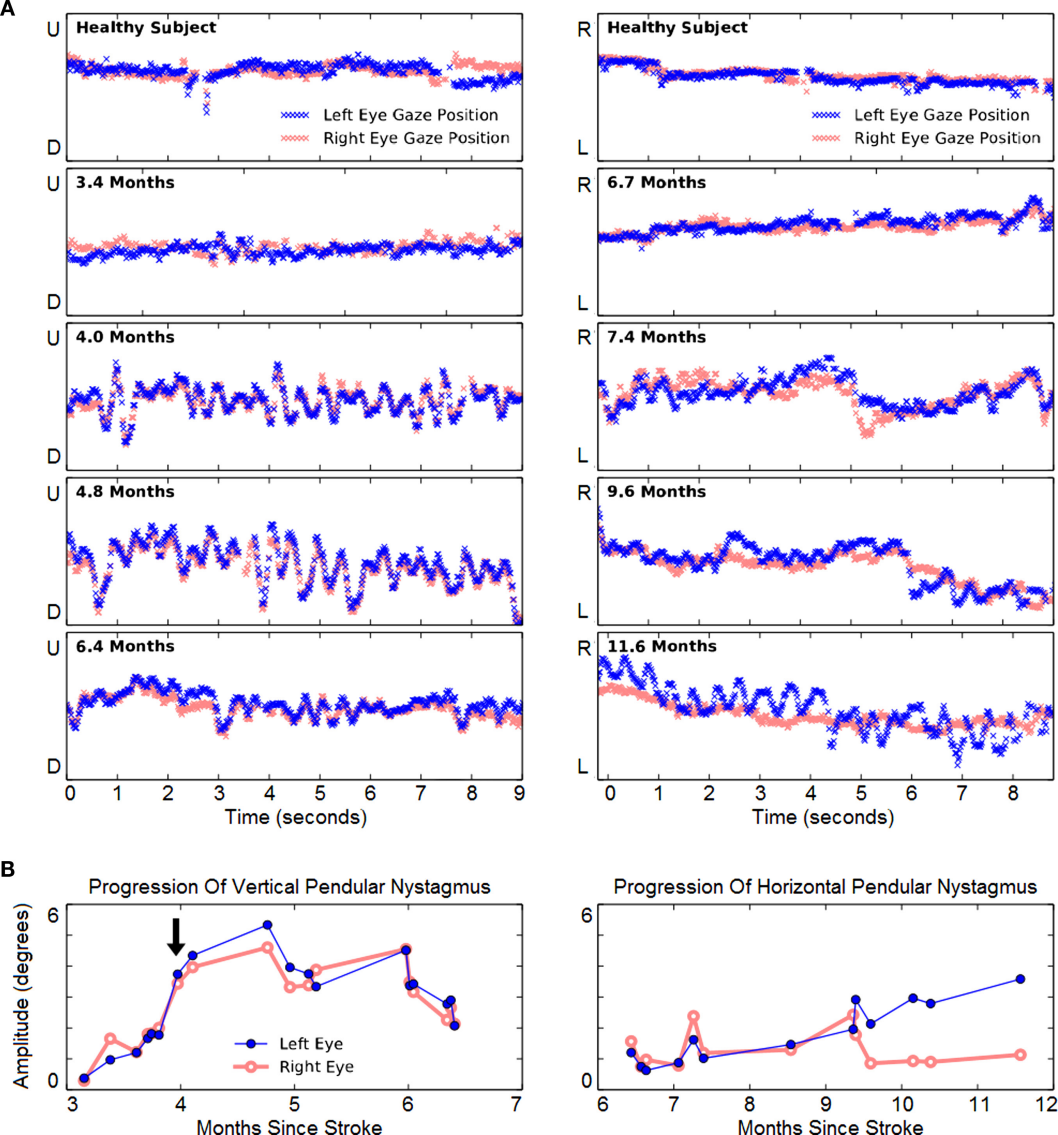
**Evolution of involuntary eye movements**. **(A)** Left panels: representative 9-s vertical gaze traces during rightward tracking. Recordings from a neurologically healthy subject (top panel) and from the case subject (lower four panels) are presented. The *Y*-axis span corresponds to 22.8° of vertical visual field. The subject initially showed mild vertical pendular nystagmus (VPN), which increased and endured until 6 mont left-eye nystagmus hs, and then improved. Right panels: representative 9-s horizontal gaze traces during downward tracking. The *Y*-axis span corresponds to 22.8° of horizontal visual field. The first evidence of horizontal pendular nystagmus (HPN) emerged in both eyes at 7.4 months. Thereafter, right-eye nystagmus was largely unchanged, but left-eye nystagmus progressively increased. **(B)** Longitudinal change of VPN (left panel) and HPN (right panel). The arrow indicates the subject’s first report of the onset of involuntary eye movements.

Vertical eye-tracking records during a horizontal task (left panels of Figure 4) revealed oscillations at 3.4 months. At this early stage, the oscillations were of similar amplitude to spontaneous vertical deviations observed in a healthy subject performing the same task (top left panel). Although they were not apparent to the patient, the amplitude of oscillations continued to increase steadily from months 3 to 4 after hemorrhage. The amplitude of the VPN then increased and peaked at 4.8 months. By 6.4 months it was much reduced, though it did not disappear. Throughout this period there was a significant increase in the amplitude of VPN in both left (*r* = 0.49, *p* = 0.041, *N* = 18) and right eye (*r* = 0.48, *p* = 0.046, *N* = 18).

In subsequent months, HPN also developed (right panels of Figure 4). When vertical-tracking measurements began at 6.7 months, there was little horizontal movement when tracking vertical movement. From 9.4 months onward, HPN was increasingly noticeable in the left eye. Over all, its increasing trend was significant in the left eye (*r* = 0.94, *p* = 2 × 10^–6^, *N* = 13) but not in the right (*r* = −0.03, *p* = 0.933, *N* = 13) as indeed the right-eye oscillations appeared to resolve abruptly at 9.6 months. The patient engaged exclusively in vertical pursuit tests in the period from 6 to 9 months, while completing 501 min over 140 sessions.

## Discussion

Asymmetrical limitations in left- and right-eye movements resulted in diplopia, the chief complaint of the patient at the beginning of this study. The eye-tracking task enabled quantification of the improvement in voluntary eye movements over the course of 4 months. The record of his improvement in range-of-movement, as well as the reduction of the right-eye delay in initiating tracking, paralleled his impression that the diplopia had improved over this period. Indeed, by the end of month 6, the patient no longer felt the need to close his right eye to compensate for the diplopia, and his range of gaze in both eyes and both directions had improved.

The patient developed VPN and HPN as well as palatal tremor beginning 4 months after pontine hemorrhage. The vertical movements and palatal tremor preceded the horizontal movements by about 4 months. This separation in time between the emergence of vertical and horizontal nystagmus supports the conception of independent generators in the inferior olive. There are too few reports of this condition to conclude whether the particular order in which our patient experienced the symptoms is typical. His vertical nystagmus was conjugate and of roughly equal amplitude between the two eyes, whereas his horizontal nystagmus was of much greater amplitude in the left eye. It is expected that such details of the movement pattern are idiosyncratic to each patient (17).

This is the first prospective measurement of the development of pendular nystagmus. The results reveal presymptomatic oscillatory eye movements whose amplitude grew steadily over the course of 3 weeks, prior to a sharp increase in amplitude that coincided with the patient’s first report of oscillopsia. As the inferior olive becomes hypertrophic (a process that can also be detected in medical imaging prior to symptoms being reported) (24), it is possible that measurable oscillations are present, but imperceptible to the patient. Thus, regular evaluation of eye-tracking may be able to detect the emergence of nystagmus in its early stages and provide an early opportunity for therapeutic intervention.

The eye-tracking application, called OptokineSys, was not designed with eye-movement disorders in mind; rather, it was designed for optokinetic vision testing (25, 26). Hence, it was not optimized to measure the most apparent parameters of gaze limitation, such as the limits of absolute horizontal gaze position. Nor was our equipment able to isolate certain details that might be expected to accompany pendular nystagmus, such as saccadic intrusions or involuntary torsional eye movements. The phenomenon it measures directly, smooth pursuit eye movements, was related only indirectly to the patient’s gaze disorders. Its use in this patient was serendipitous, driven and sustained by the fact that the patient found it motivating and enjoyable. Its success in this regard can be attributed to two factors. First, it provided real-time feedback in the form of the patient’s favorite music. Second, it yielded consistently different tracking-quality scores in different measurement conditions (combinations of eye tracked and direction of stimulus motion): in this way it provided the patient with clear targets for improvement, in measures that (albeit indirectly) reliably reflected the severity and nature of his gaze disorders. Therefore, despite its off-label usage, OptokineSys afforded an opportunity that would have been impossible to achieve by other available means: specifically, it provided prospective quantitative eye-movement information at frequent regular intervals in the patient’s home.

## Concluding Remarks

A novel eye-tracking methodology enabled, for the first time, detailed quantification of the onset, natural history, and improvement of impairments in voluntary eye movement after pontine hemorrhage. It also characterized prospectively for the first time, the emergence of involuntary eye movements resulting from the rare complication of hypertrophic olivary degeneration. By revealing a presymptomatic period of nystagmus, this study raises the possibility of using in-home testing of eye movements as a screening tool, which might facilitate earlier clinical evaluation. We have shown that it is possible to make longitudinal measurements of ocular motor dysfunction in a home setting, without extensive participant training or the need for expert oversight. Repeated measurements may provide the opportunity to better determine the extent of injury and the effectiveness of therapy.

## Ethics Statement

This study was carried out in accordance with the recommendations of the guidelines for human subjects research by the institutional review board of the Burke Medical Research Institute, with written informed consent from all subjects. All subjects gave written informed consent in accordance with the Declaration of Helsinki. The protocol was approved by the Burke IRB.

## Author Contributions

MS performed the measurements, analyzed the data, and wrote the manuscript. GP designed the methodology and wrote the manuscript; JC designed the methodology, performed clinical assessments, and wrote the manuscript; NJH designed the methodology, designed and implemented the software, analyzed the data, and wrote the manuscript.

## Conflict of Interest Statement

The patent application US 62/185,983b (2015, 2016) by authors NJH, GP, and JC covers the algorithm, methodology, and assembly of hardware components described here under the name “OptokineSys.” MS declares that the research was conducted in the absence of any commercial or financial relationships that could be construed as a potential conflict of interest.

## Supplementary Material

Video S1 **Clinical assessments during the year following hemorrhage**. (00:00-00:18) Horizontal Pursuit 3.0 Months. The range-of-movement is limited in both eyes when looking to the left or right 3 months after hemorrhage. Nystagmus is present in the left eye during leftward gaze and in the right eye during rightward gaze. (00:18-00:28) Horizontal Saccades 6.5 Months. Note the slowing of the saccades. (00.28-00:49) Horizontal Pursuit 6.5 months. The patient can perform conjugate horizontal tracking movements in a wider range with both eyes in both directions. Involuntary vertical eye movements are present in both eyes during pursuit. (00:49-01:01) Involuntary Eye Movements 6.5 Months. When staring straight ahead both eyes make repetitive involuntary vertical movements. (01:01-01-14) Involuntary Eye Movements 11.6 Months. When staring straight ahead, involuntary vertical movements are still present in both eyes, and horizontal pendular nystagmus is now also visible in the left eye. (01:14-01:25) Palatal Tremor 11.6 Months. This sign frequently accompanies pendular nystagmus.
